# Semaphorin 3A attenuates cardiac autonomic disorders and reduces inducible ventricular arrhythmias in rats with experimental myocardial infarction

**DOI:** 10.1186/s12872-016-0192-8

**Published:** 2016-01-19

**Authors:** Hesheng Hu, Yongli Xuan, Mei Xue, Wenjuan Cheng, Ye Wang, Xinran Li, Jie Yin, Xiaolu Li, Na Yang, Yugen Shi, Suhua Yan

**Affiliations:** Department of Cardiology, Shandong Provincial Qianfoshan Hospital, Shandong University, 250014 Jinan, China

**Keywords:** Myocardial infarction, Semaphorin 3A, Cardiac autonomic nerve, Ventricular arrhythmia

## Abstract

**Background:**

To investigate the effects of semaphorin 3A (sema 3A) on cardiac autonomic regulation and subsequent ventricular arrhythmias (VAs) in post-infarcted hearts.

**Method and results:**

In order to explore the functions of sema 3A in post-infarcted hearts, lentivirus-Sema 3A-shRNA and negative control vectors were delivered to the peri-infarcted myocardium rats respectively. Meanwhile, recombinant sema 3A and control (0.9 % NaCl solution) were injected intravenously into infarcted rats to test the therapeutic potential of sema 3A. Results indicated that levels of sema 3A were higher in post-infarcted hearts compared with sham rats. However, sema 3A silencing leaded to sympathetic hyperinnervation, increased myocardial norepinephrine (NE) content and inducible VAs. Conversely, the intravenous administration of sema 3A to infarcted rats reduced sympathetic nerve sprouting, improved cardiac autonomic regulation, and decreased the incidence of inducible VAs. However, both infarct size and cardiac function were similar among infarcted hearts.

**Conclusions:**

The upregulation and administration of sema 3A exerted beneficial effects on infarction-induced cardiac autonomic disorders by increasing cardiac electrical stability and reducing VAs. Sema 3A might be a potential therapeutic agent for cardiac autonomic abnormalities induced arrhythmias.

## Background

Despite advances in management strategies and patient education, ventricular arrhythmias (VAs) remain an unsolved problem, and the identification for patients at a high risk of sudden cardiac death due to myocardial infarction (MI) is still challenging [1, 2]. Increasing studies have identified that the neural mechanism is correlated with ventricular arrhythmogenesis [3–7]. Neural control of the heart is mediated through the parasympathetic and sympathetic branches of the autonomic nervous system, which jointly maintain the normal cardiac function and electrophysiological stability via innervation balance and functional confrontation [8]. An imbalanced autonomic nervous system, especially the reduced parasympathetic and increased sympathetic tone, has been commonly found in post-infarcted patients [9]. MI complicated by sympathovagal imbalance occurs in some patients, despite of preserved ventricular function, sufficient exercises, and the use of beta-blockers [10]. All these findings suggest that cardiac autonomic disorders complicated MI is associated with an unfavorable prognosis, therefore, further studies should be performed.

Cardiac innervation is highly plastic and changed over time at different stages of cardiovascular disease [3]. MI induces nerve reinnervations including sympathetic nerve and cholinergic nerve fibers [11], and infarction-induced nerve sprouting is mainly sympathetic nerve fibers [3, 12]. The increasing sympathetic nerve density caused by autonomic imbalance is characterized by increased sympathetic and decreased parasympathetic activity [8]. Therefore, infarction-induced cardiac autonomic abnormalities might lead to sympathetic over-activation and subsequent VAs.

Cardiac innervation is sculpted by growth factors during infarction. Nerve growth factor (NGF), a chemoattractive factor, plays a key role in sympathetic nerve sprouting and hyperinnervation [13]. Our previous study demonstrated that NGF has pleiotropic effects on infarcted hearts, and downregulation of NGF does not improve prognosis [14]. However, cardiac reinnervation is also modulated by chemorepulsive factors such as semaphoring 3A (sema 3A). Sema 3A is a secreted protein that regulates axon/dendrite growth and neuronal migration. It initiates growth cone collapse, inhibits axonal outgrowth, and plays crucial roles in neural, cardiac and peripheral vascular patterning [15]. Sema 3A-deficient mice exhibit sympathetic hyperinnervation, whereas sema 3A overexpressing mice lack sympathetic innervation in developing hearts [16]. Thus, sema 3A is an important regulatory factor that maintains the balance of cardiac autonomic nerve during heart development.

The changes in expression and function of semaphorins are correlated with the regenerative failure following nerve injury [15]. Sema 3A is upregulated during brain ischemia and spinal cord injury [17, 18]. Overexpression of sema 3A is also found in MI at the infarcted border [19]. Meanwhile, sema 3A-deficient mice were with high risk of sudden death and much more susceptible to VAs, which is characterized by a high level of sympathetic nerve density [16]. In addition, upregulating sema 3A by transfecting the sema 3A gene into the peri-infarcted zone could reduce sympathetic hyper-reinnervation and inducible VAs in post-infarcted hearts [19]. Moreover, variations in the sema 3A gene were identified in unexplained cardiac arrest patients with documented ventricular fibrillation [20]. Based on the above analyses, sema 3A expression plays an important role in cardiac innervation in heart development and diseased hearts. The importance of appropriate sema 3A expression in post-infarcted hearts is highlighted via downregulation or inhibition of myocardial sema 3A [19]. Therefore, sema 3A may be a potential therapeutic agent in sympathetic hyperinnervation and subsequent lethal VAs.

In the current study, the effects of sema 3A were investigated by regulating cardiac sema 3A expression via the local intramyocardial injection of lentiviral-mediated sema 3A shRNA and the intravenous injection of recombinant sema 3A. Our data revealed that silencing sema 3A augmented sympathetic hyperinnervation, increased myocardial NE content and inducible VAs. Conversely, the administration of exogenous sema 3A attenuated those abnormalities and protected infarcted hearts from inducible VAs.

## Methods

### Preparation of sema 3A shRNA lentiviral vector

RNA interference (RNAi) is a post-transcriptional process that is triggered by the introduction of double-stranded RNA (dsRNA), which leads to gene silencing in a sequence-specific manner. Lentiviral vectors provide a method of stably introducing exogenous DNA into cells that are difficult to transfect, allowing for the ectopic expression or silencing of genes for therapeutic or experimental purposes [21]. A small interference RNA (siRNA) design tool was used to design the RNA target sequences. Three selected siRNAs targeting different sites of the sema 3A gene were synthesized, and the corresponding DNA oligonucleotide (oligo) was cloned into a lentiviral expression vector. Then, the most effective short hairpin RNA (shRNA) target was determined by assessing the silencing efficacy in rat myocardial cells. The lentivirus expressing the optimal shRNA targeting sema 3A was then propagated and harvested using a virus packaging system (Telebio, Shanghai, China). Next, viral titers were determined using qPCR [22]. The shRNA construct (GFPi) targeting the reporter gene eGFP was included as a control. The lentivirus-sema 3A-shRNA titer was determined as 2.5 × 10^12^ vector genomes (vg)/ml, and the lentivirus-GFP titer was 1 × 10^13^ vg/ml.

### MI model and sema 3A intervention in vivo

All animal experimental procedures were approved by the Ethics Committee for Animal Studies of Shandong University, China, and conformed to the Guide for the Care and Use of Laboratory Animals published by the United States National Institutes of Health (NIH publication No. 85–23, revised 1996).

Male Wistar rats (8 weeks old, 280–300 g) were obtained from animal center of Shandong University and housed under 12 h light/dark cycles in a temperature-controlled room with free access to food and water. The left anterior descending (LAD) coronary artery was ligated to induce MI, as described previously [14]. Briefly, rats were anesthetized with 30 mg/kg of 3 % sodium pentobarbital (intraperitoneal [ip]). The heart was then exposed using a fourth intracostal left lateral thoracotomy after mechanical ventilation. The LAD artery was ligated permanently at 2 mm from its origin. Coronary occlusion was confirmed by ST elevation on a surface electrocardiogram (ECG), as well as regional pallor and stiff movement of the left ventricle (LV). Meanwhile, Masson staining was conducted to determine the infarct size of rats on the day of sacrifice. With respect to clinical importance, only rats with moderate infarct size (30 to 50 %) were enrolled (data was not shown here).

In order to knockdown sema 3A expression, 80 μl virus solution including 1.09 × 10^9^ TU/ml lentivirus-sema 3A-shRNA encoding green fluorescent protein(GFP) and containing sema 3A shRNA (MI-SiRNA group, *n* = 17) was injected intramyocardially at four sites in the peri-infarcted myocardium (~2 mm around the infarcted area), as reported previously [14]. The same amount of virus solution only encoding GFP (MI-GFP, *n* = 16) was injected to the rats in control groups. To investigate the potential therapeutic of sema 3A, the prepared recombinant sema 3A (Sino Biological Inc, China; MI-Sema group, *n* = 11; 1 mg/kg body weight) or PBS (MI-PBS group, *n* = 9) was injected intravenously weekly 3 days after coronary ligation for 4 weeks. An additional group of rats underwent only LAD ligation (MI-CON group, *n* = 9), and a group that underwent thoracotomy and pericardiotomy (*n* = 15) were used as the Sham group. After the incision was closed, the rats were allowed to recover from the anesthesia in a heated box, and were then returned to their individual cages.

### Hemodynamic measurements and electrophysiological study

Five weeks after the operation, rats were tracheotomized, intubated, ventilated mechanically, and monitored after anesthesia. A pressure-volume catheter (SPR-869, Millar, Houston, TX, USA) was inserted into the right carotid artery of rats to measure the mean arterial blood pressure (MAP). Then, the transducer was advanced from the right carotid artery into the LV to get the pressure-volume (P-V) data. LabChart Pro software (AD Instruments, Sydney, Australia) was utilized to evaluate LV end-systolic pressure (LVESP), LV end-diastolic pressure (LVEDP), the maximal slope of LV systolic pressure increment (dP/dtmax), diastolic pressure decrement (dP/dtmin), end-diastolic volume (EDV), end-systolic volume (ESV) and LV ejection fraction (EF).

In addition, an electrophysiological study was performed to evaluate the susceptibility of rats in a stable condition to VAs. The protocol used for programmed electrical stimulation (PES) was performed as reported previously [4, 23]. After monitoring the surface ECG, a second thoracotomy was carried out. PES was performed via a specially modified electrode with a needle-point inserted into the epicardial surface of the infarcted border (2 mm deep). After measuring the pacing threshold, standard PES protocols were performed as follows: burst (cycle length 100 ms, S0), single (S1), double (S2), and triple (S3) extrastimuli. The coupling interval of the last extra stimulus was decreased from 80 ms to the value of ventricular effective refractory period with 2-ms steps. The experimental protocols were completed within 10 min. Ventricular tachyarrhythmias, including ventricular tachycardia and ventricular fibrillation, were considered non-sustained when they lasted < 15 beats and sustained when they lasted > 15 beats. Ventricular arrhythmia scores were determined by the inducibility quotient of ventricular tachyarrhythmias as follows: 0, non-inducible; 1, non-sustained tachyarrhythmias induced with three extrastimuli; 2, sustained tachyarrhythmias induced with three extrastimuli; 3, non-sustained tachyarrhythmias induced with two extrastimuli; 4, sustained tachyarrhythmias induced with two extrastimuli; 5, non-sustained tachyarrhythmias induced with one extrastimulus; 6, sustained tachyarrhythmias induced with one extrastimulus; 7, tachyarrhythmias induced during a train of eight stimuli (8 × S1) at a basic cycle length of 100 ms; and 8, heart stopped before PES. The highest score was used when multiple forms of tachyarrhythmias occurred in one heart [23]. Finally, heart tissues were sampled according to corresponding experimental techniques.

### Immunohistochemistry and Masson’s trichrome staining

Immunohistochemistry and Masson’s trichrome staining were performed as described previously [14]. Briefly, paraffin sections were deparaffinized, rehydrated, incubated and then treated with citric acid buffer. After incubated with serum-free protein blocking buffer (ZSGB-BIO, Beijing, China), sections were incubated with rabbit anti-TH (tyrosine hydroxylase, 1:100; Millipore, Billerica, MA, USA), rinsed and incubated in horseradish peroxidase- (HRP-) conjugated secondary antibodies, and then counterstained with hematoxylin. Finally, the sections were mounted and examined using a microscopy. The density is expressed as the ratio of labeled nerve fiber area to total area, while papillary muscles were excluded from the study because a variable sympathetic innervation has been reported [24].

In addition, samples from the apex, mid-LV, and base were paraffin-embedded, sectioned and stained with Masson’s trichrome stain. The infarct size percentage was calculated as fibrosis area/total LV area × 100. All images were analyzed with ImageJ software ImagePro Plus 5.0 (Media Cybernetics, Bethesda, MD).

### Western blotting

Proteins were extracted from cardiac issues prepared from the infarcted border using a Nuclear and Cytoplasmic Protein Extraction Kit (Beyotime, Haimen, China). Protein concentrations were quantified using a bicinchoninic acid (BCA) protein assay kit (Beyotime). From each extract, 40 μg proteins was separated using 10 % SDS-PAGE, and then transferred to polyvinylidene fluoride (PVDF) membranes (Bio-Rad, Hercules, CA, USA). Membranes were blocked in 5 % non-fat milk, followed by incubated with anti-TH (1:1000), anti-CHAT (choline acetyltransferase, 1:1500; Millipore, Billerica, MA, USA), anti-NGF (1:1500, Epitomics, Burlingame, CA, USA), anti-sema 3A (1:1000, Abcam, Cambridge, England) or anti-GAPDH (glyceraldehyde-3-phosphate dehydrogenase, 1:3000; CoWin Bioscience, Beijing, China) antibodies. After washed with PBS, the membranes were incubated with the corresponding secondary antibodies, and images were developed using an enhanced chemiluminescence detection kit. Immunoreactive bands were visualized using a FluroChem E Imager (ProteinSimple, Santa Clara, CA, USA). The expression levels of the target proteins were measured and normalized to GAPDH.

### Real-time quantitative PCR

Total RNA was isolated from samples at infarcted border zone (3 mm zone adjacent to the infarcted area), and the mRNA expression levels of TH, CHAT, NGF, and sema 3A were assessed by real-time quantitative RT-PCR using a PrimeScript RT reagent kit (TaKaRa, Dalian, China) in a Mastercycler EP realplex detection system (Roche, Indianapolis, IN, USA) as reported previously. For each sample, GAPDH and the target genes were amplified in duplicate in separate tubes. Each measurement was performed in triplicate. Gene expression was analyzed using the 2^-∆∆CT^ method described by Livak and Schmittgen [25]. The primers for each gene used in this study were as follows:

NGF forward 5‘-TCGCTCACTCCACTATCCACTA-3’, and reverse 5‘-GACTCAACAGGGCAAGCATAC-3’; sema 3A forward 5‘-GAGTGATGTAAGAAGGGTGTTCC-3’, and reverse 5‘-CAAGTTCCTGGTCGTGGATAAG-3’; TH forward 5‘-GGCTTCTCTGACCAGGTGTATC-3’, and reverse 5‘-TAGCAATCTCTTCCGCTGTGTA-3’; CHAT forward 5‘- AGCCCCTCTGTATGAAGCAAT--3’, and reverse GGACGCCATTTTGACTATCTTT-3’; GAPDH forward 5‘-ACAGCAACAGGGTGGTGGAC-3’, and reverse 5‘-TTTGAGGGTGCAGCGAACTT-3’.

### High performance liquid chromatography (HPLC)

According to previously reported studies [26, 27], we measured norepinephrine(NE) and acetylcholine (ACh) levels in heart tissue isolated from the infarcted border using HPLC with electrochemical detection. Briefly, fresh heart samples (50 mg) were transferred immediately into perchloric acid containing isopropylhomocholine as an internal control. They were then homogenized, centrifuged, and filtered to obtain an HPLC samples. HPLC was then used to quantify Ach and NE levels.

## Statistical analyses

Data are presented as means ± standard deviations (SD). Independent *t*-tests were used to compare values between two groups. ANOVA followed by Tukey’s test was used to compare differences between more than two groups. Analyses were performed using SPSS 17.0 software (SPSS Inc., Chicago, IL, USA). A value of *P* < 0.05 was considered statistically significant.

## Results

### Sema 3A knockdown using recombinant lentivirus vectors in vitro and in vivo

**Fig. 1:**
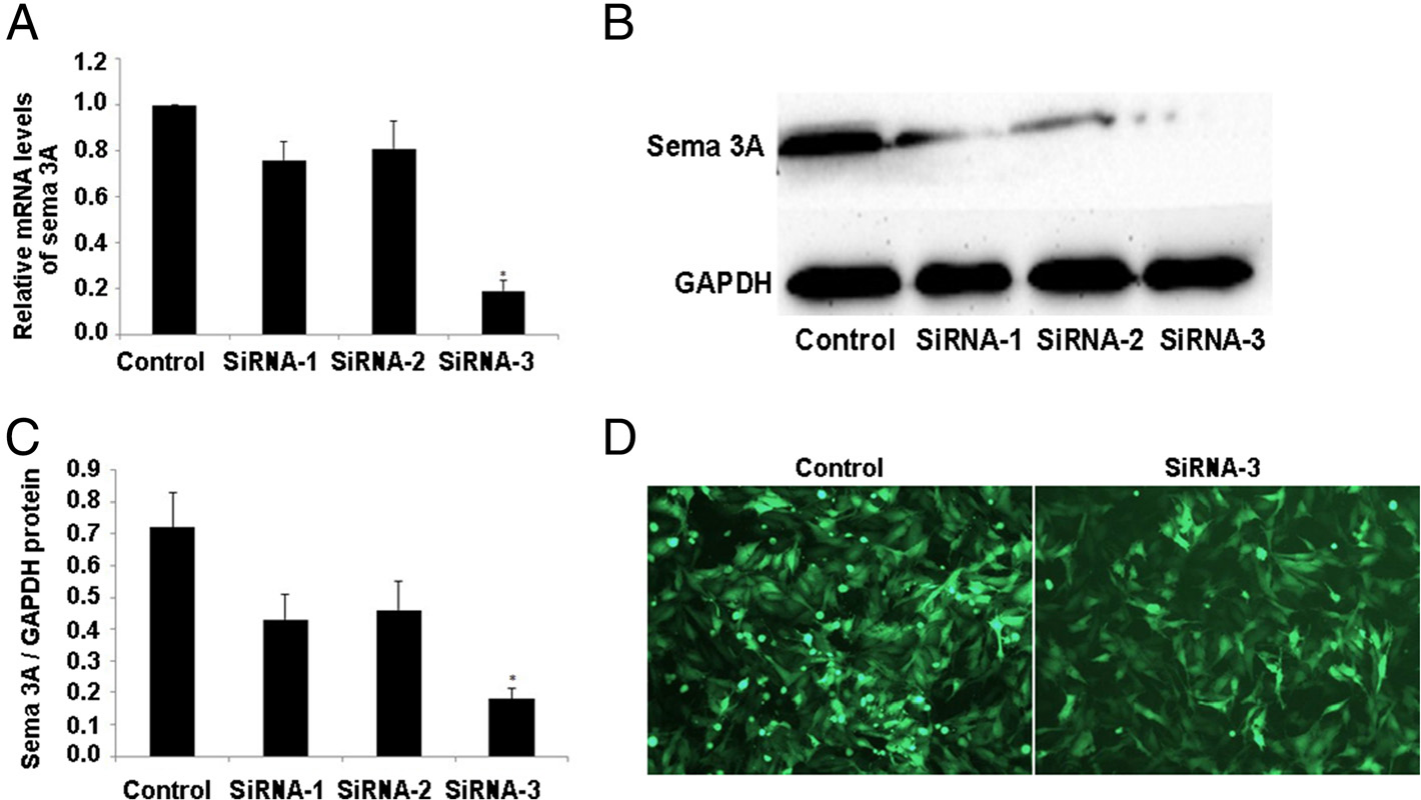
The effectiveness of sema 3A-siRNA as determined by reduced protein and mRNA levels in vitro. **a** RT-PCR demonstrating sema 3A mRNA expression in rat myocardial cells transfected with sema 3A-shRNA 1 (*SiRNA-1*), 2 (*SiRNA-2*), and 3 (*SiRNA-3*), or PBS-control (*Control*) (*P < 0.01 vs. SiRNA-1). **b**, **c** Western blotting showing the expression of sema 3A in rat myocardial cells in various groups. (*P < 0.01 vs. SiRNA-1). **d** Image showing the effect of shRNA-3 in cultured rat myocardial cells using fluorescence microscopy

**Fig. 2:**
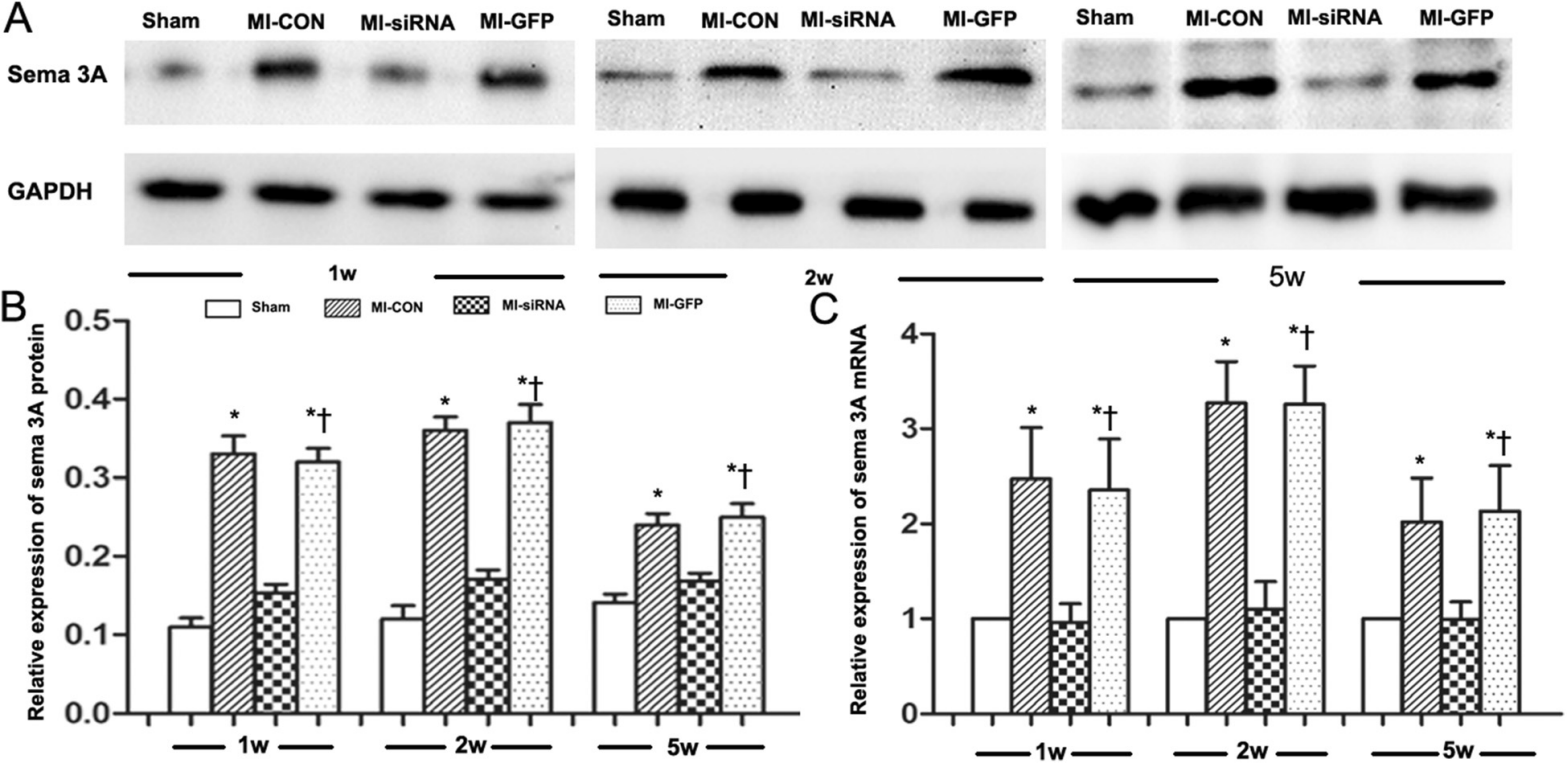
The time-course expression of sema 3A in myocardial infarction (MI) models after lentivirus-sema 3A-shRNA treatment. **a**, **b** Western blotting was conducted to detect the effects of lentivirus-sema 3A-shRNA on the protein level of sema 3A (95 KDa) at various time points (1, 2, and 5 weeks). The relative expression of sema 3A was normalized to GAPDH (36 KDa). **c** Relative mRNA expression of *sema 3*A was detected using real-time quantitative RT-PCR. The relative gene expression of sema 3A was analyzed using the 2^-ΔΔCT^ method. Data are presented as means ± SD. *P < 0.05 vs. sham group. †*P* < 0.05 vs. MI-siRNA group

The mRNA and protein expression of sema 3A were assessed in a rat myocardial cell line to identify the knockdown efficacy of different siRNAs (Fig. 1). The results suggested that the sequence 5‘-GGTGTTCCTTGGTCCATATGC-3’ resulted in the most effective knockdown of sema 3A in vitro. Therefore, this sequence was packaged and used for in vivo studies. Both mRNA and protein levels of sema 3A were higher in the MI-CON group than those in the Sham group at 1, 2, and 5 weeks after infarction (*P* < 0.01, Fig. 2). In addition, compared with the MI-GFP group, the expression of sema 3A was significantly lower in the MI-SiRNA group (*P* < 0.01, Fig. 2).

### Effects of Sema 3A on inducible VAs, infarct size, and hemodynamics in post-infarcted hearts

**Fig. 3:**
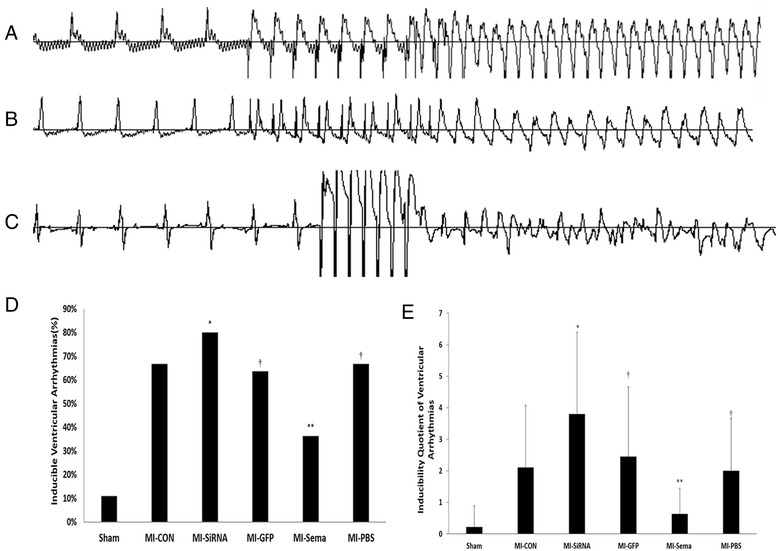
Various ventricular arrhythmias induced by programmed electrical stimulation in infarcted rats. **a** Eight basic stimuli (S0) at cycle lengths of 100 ms and two extra stimuli (S1 and S2) induced monomorphic ventricular tachycardia (VT). **b** Eight basic stimuli (S0) and two extra stimuli (S1 and S2) induced polymorphic ventricular tachycardia. **c** Seven basic stimuli (S0) induced ventricular fibrillation (Vf). **d** The percentage of inducible total ventricular arrhythmias/ stimulation times.* *P* < 0.05 vs. MI GFP group; **P < 0.01 vs. MI-PBS group; †*P* > 0.05 vs. MI-CON group. **e** Inducibility quotient of induced ventricular arrhythmias 5 weeks after infarction. * *P* < 0.01 vs. MI GFP group; **P < 0.01 vs. MI-PBS group, †*P* > 0.05 vs. MI-CON group

There was no significant difference in the infarct size and hemodynamic data among the MI groups (Table 1). To assess the incidence of potential VAs, we designed an electrophysiological study to assess the cardiac electrical stability. Ventricular tachyarrhythmias were inducible by programmed stimulation in infarcted rats as showed in Fig. 3a–c. The percentage of inducible ventricular arrhythmia is negatively correlated with the expression level of sema 3A (Fig. 3d). The arrhythmia scores for sham rats were nearly 0 (Fig. 3e). Silencing sema 3A significantly increased the inducibility of ventricular tachyarrhythmia in infarcted rats compared with counterpart vehicle treatment; in contrast, sema 3A administration significantly decreased the inducibility of ventricular tachyarrhythmia (both *p* < 0.05) (Fig. 3e). Therefore, exogenous sema 3A could stabilize cardiac electrical activity and reduce the incidence of VAs.

**Table 1:** Hemodynamic data based on pressure-volume and infarct size 5 weeks after MI

	Sham	MI-CON	MI-GFP	MI-SiRNA	MI-Sema	MI-PBS
n	9	9	10	11	11	9
Infarct size (%)		42.7 ± 5.1	42.9 ± 4.7	44.3 ± 5.2	43.5 ± 4.2^**^	41.3 ± 4.9
HR (beats/min)	419.2 ± 19.1	424.5 ± 17.2^*^	421.4 ± 21.2	427.3 ± 19.2	419.2 ± 19.1^**^	420.1 ± 19.2
MAP (mmHg)	109.2 ± 3.7	92.4 ± 6.4^*^	93.2 ± 7.9	89.7 ± 8.1	92.7 ± 7.3^**^	93.7 ± 7.7
LVDSP (mmHg)	2.92 ± 0.97	6.3 ± 1.2^*^	5.8 ± 1.4	5.7 ± 1.6	6.1 ± 1.9^**^	5.9 ± 1.4
LVESP (mmHg)	119.3 ± 9.2	91.2 ± 5.9^*^	89.2 ± 6.1	90.2 ± 7.1^**^	88.3 ± 6.8^**^	91.4 ± 7.1
EDV (μl)	309.4 ± 21.3	483.2 ± 33.7^*^	467.6 ± 41.4	475.1 ± 48.1^**^	471.3 ± 47.4^**^	469.3 ± 45.3
ESV (μl)	106.1 ± 114	293.4 ± 19.4^*^	289.2 ± 23.1	291.2 ± 24.1^**^	289.4 ± 27.3^**^	285.7 ± 24.2^**^
EF (%)	61.2 ± 1.7	34.7 ± 0.7^*^	35.3 ± 06	34.9 ± 0.8	34.2 ± 0.9^**^	33.9 ± 1.0
dP/dtmax (mmHg/s)	5517.4 ± 129	3696.2 ± 90.2^*^	3712.7 ± 91.8	3637.4 ± 83.7	3707.2 ± 73.1^**^	3684.3 ± 80.5
dP/dtmin (mmHg/s)	−3989.1 ± 147	−2379.4 ± 77.5^*^	−2394.7 ± 90.3	−2427.4 ± 84.3	−2312.5 ± 74.9^**^	−2419.4 ± 94.2

Values are presented as means ± SD. HR, heart rate; *MAP* mean arterial pressure, *LVEDP* LV end-diastolic pressure, *LVESP* LV end-systolic pressure, *EDV* end-diastolic volume, *ESV* end-systolic volume, *EF* ejection fraction, *dP/dtmax and dP/dtmin* maximal slope of the systolic pressure increment and the diastolic pressure decrement, respectively.^*^ *P* < 0.05 vs. sham group, ^**^ *P* > 0.05 among various MI groups

### Effects of silencing Sema 3A on sympathetic hyperinnervation, ratio of TH/CHAT and myocardial NE level

**Fig. 4:**
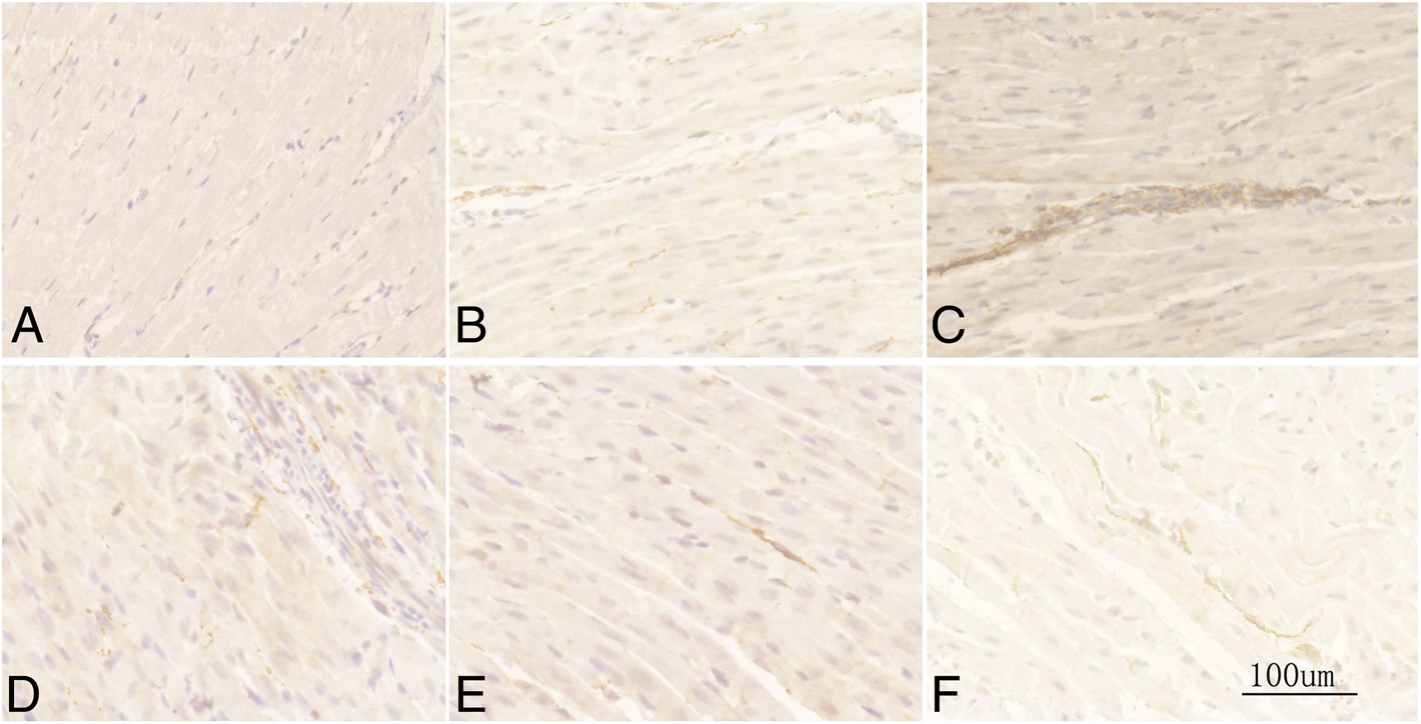
Histological study of cardiac nerve fibers at the infarcted border zone in sham-operated and infarcted hearts. Immunohistochemistry staining for tyrosine hydroxylase (magnification × 200): (**a**), MI-CON (**b**), MI-SiRNA (**c**), MI-GFP (**d**), MI-PBS (**e**), and MI-Sema (**f**) groups

**Fig. 5:**
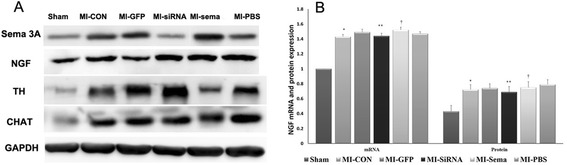
The effects of sema 3A on the expression levels of NGF, TH and CHAT. **a** Representative western blots showing sema 3A (95 KDa), NGF (27 KDa), TH (58 KDa), CHAT (72KDa), and GAPDH (36 KDa) expression in various groups. **b** MI resulted in the increase of NGF mRNA and protein expression in the MI-CON group compared with the sham group (*P < 0.05). Both were similar in various MI groups. **P > 0.05 vs. MI-GFP group; †*P* > 0.05 vs. MI-PBS group

As shown in Figs. 4 and 5 and Table 2, MI caused nerve sprouting and sympathetic neural remodeling in the MI-CON and MI-GFP groups, and the TH-positive nerve densities were increased at the infarcted border. Meanwhile, the protein expression of TH and CHAT was higher in the MI-CON and MI-GFP groups. However, a higher TH-positive nerve density was detected in the MI-SiRNA group compared with the MI-GFP group (Fig. 4 and Table 2). Sema 3A silencing increased mRNA and protein levels of TH at the infarcted border (Table 2) but did not affected CHAT expression. Thus, the ratio of TH/CHAT protein was higher in MI-SiRNA group compared with the MI-CON and MI-GFP groups (Table 2).

**Table 2:** Effects of sema 3A on autonomic abnormalities 5 weeks after infarction

		Sham	MI-CON	MI-GFP	MI-SiRNA	MI-Sema	MI-PBS
1-RT-PCR (target mRNA /GAPDH)	TH	1	2.93 ± 0.21	2.87 ± 0.18	3.82 ± 0.34^*^	1.84 ± 0.14^**^	3.12 ± 0.23^***^
	CHAT	1	1.66 ± 0.14	1.71 ± 0.17	1.67 ± 0.16	1.61 ± 0.14^****^	1.72 ± 0.17
2- IHC (um^2^/mm^2^)	TH-positive	1281 ± 75.1	3589 ± 278.1	3539 ± 176	4707 ± 193.2^*^	2330 ± 162.4^**^	3564 ± 118.9^***^
	Sema 3A protein	0.14 ± 0.02	0.35 ± 0.03	0.33 ± 0.025	0.167 ± 0.02^*^	0.44 ± 0.03^**^	0.31 ± 0.02^***^
3- WB (target protein/GAPDH)	TH protein	0.19 ± 0.03	0.45 ± 0.035	0.47 ± 0.029	0.67 ± 0.027^*^	0.35 ± 0.014^**^	0.48 ± 0.03^***^
	CHAT protein	0.085 ± 0.087	0.174 ± 0.013	0.174 ± 0.01	0.173 ± 0.011	0.167 ± 0.011^**^	0.179 ± 0.09
	TH/CHAT	2.18 ± 0.25	2.63 ± 0.26	2.67 ± 0.19	3.77 ± 0.20^*^	2.66 ± 0.18^**^	2.10 ± 0.10^***^
4- HPLC (pmol/mg)	NE	2.1 ± 0.12	4.3 ± 0.29	4.1 ± 0.31	5.1 ± 0.27^*^	2.9 ± 0.26^**^	3.9 ± 0.27^***^
	ACh	2.9 ± 0.26	3.9 ± 0.23	3.8 ± 0.27	3.5 ± 0.24	3.6 ± 0.28^****^	3.8 ± 0.27

(1) RT-PCR was used to assess the mRNA expression of *TH*, and *CHAT* in various groups. Sema 3A silencing increased the expression of *GAP 43* and *TH* compared with the MI-GFP group (*P* < 0.05). However, the administration of sema 3A reduced the expression in *TH* compared with the MI-PBS group (*P* < 0.05). *CHAT* mRNA expression was comparable between various MI groups (*P* > 0.05). (2) Immunohistochemistry results revealed that the density of TH- and GAP 43- positive nerve fibers were higher in sema 3A-silenced infarcted-hearts compared with the MI-GFP group (both *P* < 0.05). Recombinant sema 3A reduced the number of TH- and GAP 43- positive nerve fibers compared with the MI-PBS group (both *P* < 0.01). There was no difference in the density of TH- nerve fibers among the MI-CON, MI-GFP, and MI-PBS groups (all *P* > 0.05). (3) Western blot (WB) showing increased protein expression of TH and CHAT in the MI-CON group compared with the sham group (*P* < 0.05). Sema 3A silencing increased the expression of TH protein, whereas sema 3A administration reduced TH protein expression. CHAT protein expression was comparable among the various MI groups. However, the ratio of TH/CHAT protein expression was increased in the MI-SiRNA group compared with the MI-GFP group (*P* < 0.05). The ratio was lower in the MI-Sema group compared with the MI-PBS group (*P* = 0.021), but there was no significant difference between the MI-Sema and sham groups (*P* = 0.34). (4) HPLC was used to assess local sympathetic and parasympathetic activity. MI resulted in increased myocardial NE and Ach levels in the MI-CON group compared with the sham group (both *P* < 0.05). NE content was increased in the MI-SiRNA group compared with the MI-GFP group (*P* = 0.007). In addition, NE content was reduced in the MI-Sema group compared with the MI-PBS group (*P* = 0.024). However, myocardial Ach levels were similar in the various infarcted groups (*P* > 0.05). ^*^ *P* < 0.05 vs. MI-GFP group, ^**^ *P* < 0.05 vs. MI-SiRNA group, ^***^ *P* < 0.05 vs. MI-sema group, ^****^ *P* > 0.05 among various MI groups

Myocardial NE and ACh were measured to evaluate autonomic nerve function. Both myocardial NE and ACh levels at the infarcted border were higher in the MI-CON and MI-GFP groups compared with the sham group. After sema 3A silencing, myocardial NE level was higher than that in the MI-GFP group. However, there were no significant differences of ACh levels between the MI-SiRNA and MI-GFP groups (Table 2).

### Sema 3A administration promoted cardiac innervation and function

To explore the potential therapeutic effects of sema 3A in infarcted hearts, recombinant sema 3A was injected as described previously [28]. Data revealed that intravenously administrated sema 3A dramatically decreased the sympathetic nerve density nerve fibers, accompanied by decreased protein and mRNA levels of TH and decreased TH/CHAT ratio (Fig. 3 and Table 2). In addition, as is shown in Fig. 5, knockdown or overexpression of sema 3A in infarcted rats did not seem to affect CHAT level, supported by the fact that neural remodeling post-infarction is mostly characterized by sympathetic hyperinnervation. Therefore, the alleviated TH/CHAT ratio by sema 3A is mainly through the downregulation of TH.

Myocardial NE levels were lower in the MI-Sema group compared with the MI-PBS group. However, there was no significant difference in myocardial ACh levels between the MI-PBS and MI-Sema groups (all *P* > 0.05). Moreover, both the mRNA and protein levels of NGF were comparable among the MI groups (Fig. 5), which suggested that sema 3A affected the post-infarcted cardiac autonomic nerve independent of NGF.

## Discussion

Increased sympathetic tone and densities are important in the generation of VAs and subsequently sudden cardiac death. MI results in abnormal cardiac autonomic regulation in terms of both neural distribution and function. The MI-complicated abnormalities might cause unfavorable prognosis such as lethal VAs and sudden cardiac death [8, 29, 30]. The current study revealed that silencing sema 3A enhanced the abnormalities of cardiac autonomic regulation and increased the incidence of inducible VAs in post-infarcted hearts. Moreover, overexpression of sema 3A reduced sympathetic nerve sprouting, ameliorated cardiac autonomic imbalance, and decreased the incidence of inducible VAs. Infarct size and cardiac function were similar among the MI groups. Therefore, sema 3A has therapeutic potential for post-infarcted cardiac autonomic abnormalities.

Normal cardiac function and rhythm are maintained by balancing the actions of sympathetic and parasympathetic inputs to the heart [31]. The electrical and contractile activities of myocardium are modulated by the release of NE from sympathetic neurons and the secretion of ACh from parasympathetic neurons. However, the normal balance between cardiac autonomic neurons is disrupted by MI, which leads to increased sympathetic and decreased parasympathetic transmission in the heart [32]. Neurotrophins, such as sema 3A and NGF, regulate axonal growth, synaptic plasticity, survival, differentiation, myelination, and nerve patterning during both cardiac development and diseased pathologies [13, 16, 33–35].

Sema 3A is a neural chemorepellent as an axon guidance molecule that plays important roles in the development of the nervous system and axon growth [15]. Appropriate sema 3A expression in heart is required for sympathetic innervation patterning. Both sema 3A silencing and sema 3A overexpressing exhibited disrupted innervation patterning in mice [16]. In post-infarcted hearts, the lentivirus-mediated overexpression of sema 3A in the infarcted zone alleviated sympathetic hyper-reinnervation [19]. The increased expression of sema 3A in heart failure might partially account for the cardiac sympathetic denervation [36]. Consistent with the previous study [19], sema 3A expression was increased in the myocardium of MI rats. Besides, the mRNA and protein expression levels of TH were significantly higher in sema 3A-silenced rats compared with the MI-GFP and MI-CON groups. These results suggest that the upregulation of sema 3A might partially suppress sympathetic nerve sprouting and subsequently decrease sympathetic nerve expression.

Previous studies revealed an increased expression of CHAT mRNA and protein in MI [11]. Nevertheless, in the current study, the ratio of TH/CHAT protein was higher in MI-SiRNA group than that in MI-CON and MI-GFP groups. Consistently, in the MI-SiRNA group, higher levels of NE were detected accompanied with a higher arrhythmic score and an increased incidence of inducible VAs. Previous studies revealed that sympathetic hyperinnervation and activation were correlated with a high incidence of lethal VAs and sudden cardiac death in post-infarcted hearts [5, 7, 8, 30]. In addition, pro-arrhythmia effects were reported in sema 3A-related neural remodeling animals [16, 19]. Recently, Nakano et al. demonstrated that a non-synonymous polymorphism in sema 3A was correlated with human unexplained cardiac arrest and ventricular fibrillation with inappropriate innervation patterning [20]. Taken together, these data suggest that sema 3A might play a critical role in maintaining cardiac electrical stability by preserving normal cardiac innervation in diseased hearts.

In damaged neural tissues, upregulated sema 3A could hinder neuroregeneration and remyelination [15]. Overexpression of sema 3A in the infarcted border via local myocardial gene transduction reduces sympathetic hyperinnervation and inducible VAs in post-infarcted hearts [19]. In our study, sema 3A expression was increased in vivo by intravenously injecting recombinant sema 3A. Five weeks after infarction, TH-positive nerve fibers, as well as its mRNA and protein levels, were all decreased in the MI-Sema group. However, there was no significant difference in the expression of CHAT and myocardial ACh levels between the MI-control and MI-Sema groups. The protein ratio of TH/CHAT was reduced to relatively normal levels, similar to that in the sham group (*P* = 0.34), via downregulating TH expression by sema 3A. The incidence of inducible VAs and myocardial NE levels were also lower in the MI-Sema group compared with the MI-CON and MI-PBS groups. Sympathetic overactivity complicated MI leads to an increase of NE concentration which encourages the early depolarization (EAD) and delayed afterdepolarization (DAD) by affecting influx and repolarization potassium current, and then trigger arrhythmia [37]. Moreover, NE may cause focal vasoconstriction and myocardial ischemia which facilitates- arrhythmogenesis [38]. Sema 3A suppresses the expressions and functions of myocardial transient outward current (Ito) and inward rectifier current (IK1) channels. Sema 3A ameliorates electrical remodeling in post-infarcted heart which is partly related with the inhibition of sympathetic nerve sprouting [39]. All these studies indicate that sympathetic innervation is also closely related to electrical homogeneity. Consistently, overexpression of sema 3A decreased sympathetic nerve sprouting activity and nerve density, improved TH/CHAT ratio and further reduced myocardial NE level according to our results. These effects may contribute to the increased cardiac electrical stability, resulting in reduced inducible VAs.

Infarct size and cardiac dysfunction are predictive factors of VAs in post-infarcted-hearts [40]. This current study revealed that these two factors were similar among MI groups, suggesting that sema 3A affected cardiac rhythm independent of infarct size and cardiac function in post-infarcted hearts. Sema 3A inhibits NGF-induced nociceptive afferent sprouting in spinal cords of adult rats [41]. However, both sema 3A silencing and overexpression did not alter the up-regulated NGF mRNA and protein in post-infarcted hearts. Cardiac innervation patterning is strictly controlled by the balance between NGF and sema 3A [16]. Treatment with exogenous sema 3A might alleviate the infarction-induced imbalance between NGF and sema 3A and suppress nerve sprouting, especially the sympathetic nerve. Furthermore, both sema 3A deficiency and supplementation did not alter the expression of CHAT protein and mRNA levels. Therefore, sema 3A treatment improved cardiac autonomic abnormalities mainly by affecting the sympathetic nerve.

### Limitations

Coronary arteriosclerosis is the main cause of clinical MI in patients. In the current study, we ligated the left anterior descending coronary artery and created a MI model that was different from the clinical setting. Moreover, the different infarct sizes (small or large) and sites (anterior, inferior, or posterior wall) might partly affect the expression of neurotrophic factors and ventricular arrhythmogenesis. However, autonomic nerve function can be affected by animal emotions and surroundings. Clinically, heart rate variability (HBV), baroreflex sensitivity (BRS), and heart rate recovery (HRR) are used frequently to evaluate autonomic nerve function [42]. Myocaridial NE and ACh levels are also measured to assess nerve function. Nevertheless, additional studies are necessary to explore the mechanisms of effects of sema 3A on post-infarcted neural remodeling and its relationship with cardiac autonomic function. Such studies will provide further insights into sema 3A as a therapeutic target for autonomic abnormalities complicated cardiac electrical instability.

## Conclusion

MI results in nerve injury and upregulation of the neuronal regulator sema 3A. Endogenous sema 3A partially inhibits nerve sprouting, whereas the downregulation of sema 3A aggravates the cardiac autonomic disorders and increases the potential of lethal VAs in post-infarcted hearts. Intravenous injection of sema 3A improves the autonomic abnormalities at the levels of both innervation and nerve function, mainly sympathetic nerve, and subsequently increases cardiac electrical stability and reduces inducible VAs. Therefore, sema 3A might be a therapeutic target for autonomic disorders induced VAs in post-infarcted hearts.

## Abbreviations

sema 3A: Semaphorin 3A
VAs: Ventricular arrhythmias
NE: Norepinephrine
MI: Myocardial infarction
NGF: Nerve growth factor
LV: Left ventricle.
GFP: Green fluorescent protein
RNAi: RNA interference
DsRNA: Double-stranded RNA
siRNA: Small interference RNA
oligo: Oligonucleotide
shRNA: Short hairpin RNA
BCA: Bicinchoninic acid
PVDF: Polyvinylidene fluoride
LAD: Left anterior descending
ECG: Electrocardiogram
PES: Programmed electrical stimulation
GAPDH: Glyceraldehyde-3-phosphate dehydrogenase
TH: Tyrosine hydroxylase
MAP: Mean arterial blood pressure
P-V: Pressure-volume
LVESP: LV end-systolic pressure
LVEDP: LV end-diastolic pressure
dP/dtmax: Systolic pressure increment
dP/dtmin: Diastolic pressure decrement
EDV: End-diastolic volume
ESV: End-systolic volume
EF: Ejection fraction
CHAT: Choline acetyltransferase
HPLC: High performance liquid chromatography
Ach: Acetylcholine
Ito: Transient outward current
IK1: Inward rectifier current
HBV: Heart rate variability
BRS: Baroreflex sensitivity
HRR: Heart rate recovery
